# Supplementary material for: A phosphorylation-based switch controls TAA1-mediated auxin biosynthesis in plants
Source: Nat Commun. 2020 Feb 3;11:679. doi: 10.1038/s41467-020-14395-w (PMC6997161; doi:10.1038/s41467-020-14395-w)
Supplement: Supplementary file 1 — Supplementary Information [file 41467_2020_14395_MOESM1_ESM.pdf]

Supplementary information for:

A phosphorylation-based switch controls TAA1-mediated auxin biosynthesis in plants

Q. Wang, G. Qin, M. Cao, R. Chen, Y. He, L. Yang, Z. Zeng, Y. Yu, Y. Gu, W. Xing, A. Tao, and T. Xu [#]

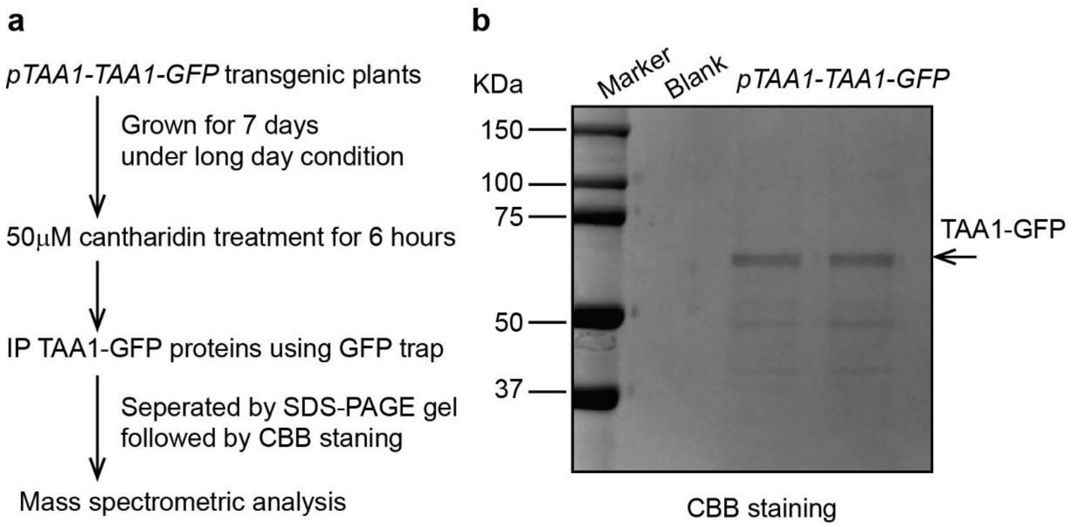

Supplementary Figure 1. Immuno-precipitation of *in vivo* TAA1-GFP proteins for mass spectrometric analysis.

(**a**) Procedure for collection of TAA1-GFP proteins.

(**b**) Image of SDS-PAGE gel stained by Coomassie brilliant blue (CBB). Immuno-precipitation result showed a clear band of TAA1-GFP proteins as the arrow indicated.

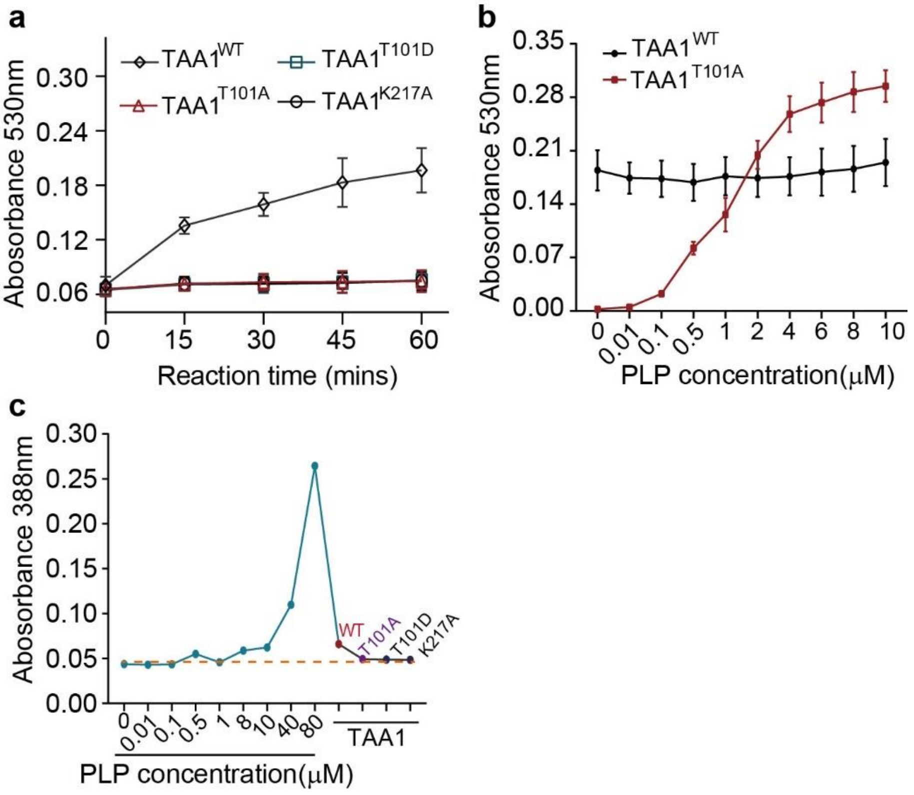

Supplementary Figure 2. T101 mutation influences TAA1 binding with PLP.

(**a**) Transaminase activity detected by measuring IPA production with Salkowski reagent (see methods). Transaminase catalytic reactions were performed in the reaction reagent without PLP using about 2.5 μg recombinant TAA1-His proteins. The IPA products were measured by reading the absorbance at 530 nm at different time points. Only *E.coli*-purified $TAA1^{WT}$ exhibited normal enzymatic activity without exogenous PLP supply. Values denoted the mean ± s.d. (n=three biological independent repeats).

(**b**) Transaminase activity detected by measuring IPA production with Salkowski reagent. Transaminase catalytic reactions were performed in the reaction reagent with different concentrations of PLP. *E.coli*-purified $TAA1^{T101A}$ showed increased enzymatic activity when adding sufficient PLP. Values denoted the mean ± s.d. (n=three biological independent repeats).

(**c**) PLP detection through measuring the absorbance at 388 nm (see method). The *E.coli*-purified $TAA1^{WT/\ T101A/T101D/\ K217A}$ proteins were denatured at 95°C to release PLP from proteins. The supernatant containing PLP was obtained by centrifuging and used for PLP

detection by reading absorbance at 388 nm (see methods). The data showed that only *E.coli*-purified TAA1^{WT} carried a certain amount of PLP from *E.coli*. Three repeats showed similar results.

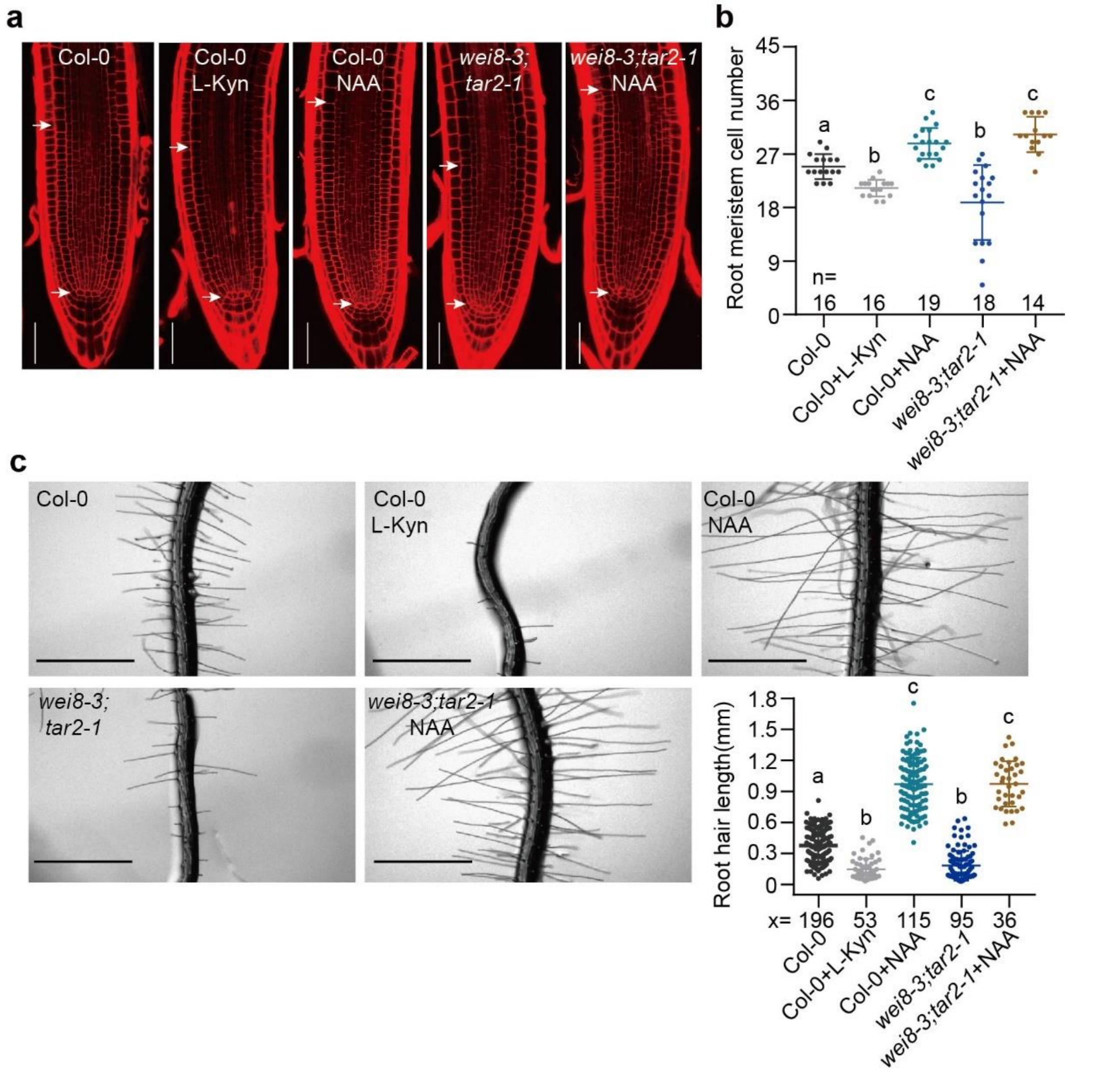

Supplementary Figure 3. Auxin regulates root development.

(**a**) Representative pictures of root apical meristem from 5-day-old seedlings. White arrows show meristem zone, scale bar, 50 μm. Around 3.19% (3/94: 3 among 94 seedlings showed strong phenotype) of *wei8-3;tar2-1* displayed collapsed root meristem.

(**b**) Quantification of root meristem size in (a). n denotes number of independent seedlings.

(**c**) Representative pictures of root hair from 5-day-old seedlings and quantification data. Col-0: n=20; Col-0+NAA: n=8; Col-0+L-Kyn: n=14; *wei8-3;tar2-1*: n=13; *wei8-3;tar2-1*+NAA: n=3. n denotes number of independent seedlings and x represents number of root hairs used for quantification. Scale bar, 1 mm. 20 nM NAA (sigma N0640) or 1.5 μM L-Kyn (sigma K3750) was added into medium for treatment. One-way ANOVA with

Tukey multiple comparisons test was used in (b) and (c). Different letters represent significant difference between each other, $P<0.05$(b), $P<0.0001$(c).

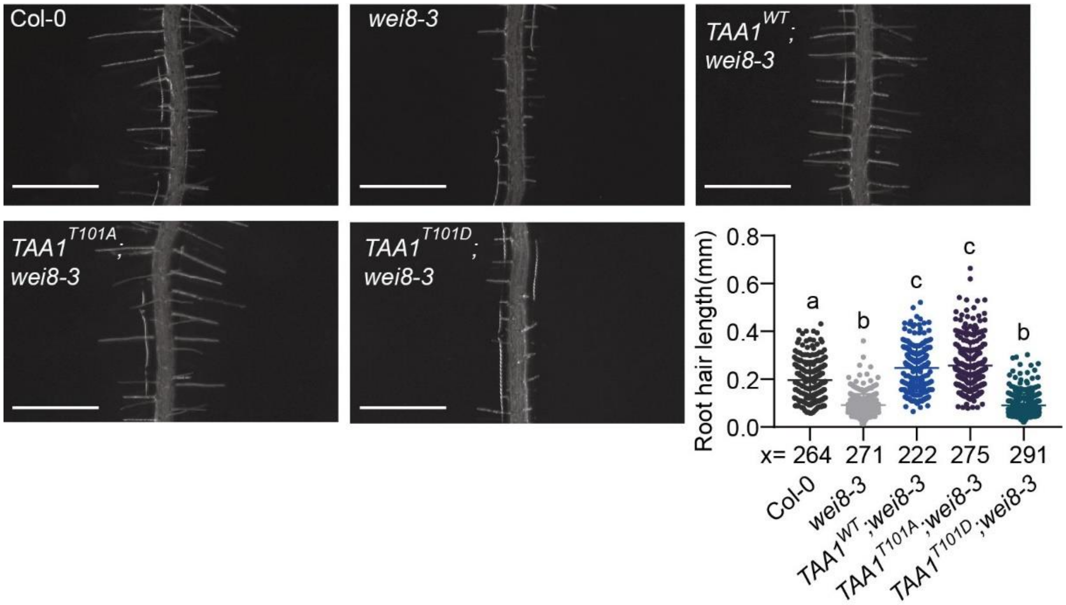

Supplementary Figure 4. T101 of TAA1 is essential for root hair development. Representative pictures of root hair from 5-day-old seedlings and quantification data. Col-0: n=20; *wei8-3*: n=21; *TAA1*^{WT};*wei8-3*: n=19; *TAA1*^{T101D};*wei8-3*: n=20; *TAA1*^{T101A};*wei8-3*: n=35. n denotes number of independent seedlings and x represents number of root hairs used for quantification. Two independent lines of each transgenic plant showed similar results. Scale bar, 0.5 mm. One-way ANOVA with Tukey multiple comparisons test was used. Different letters represent significant difference between each other, P<0.0001.

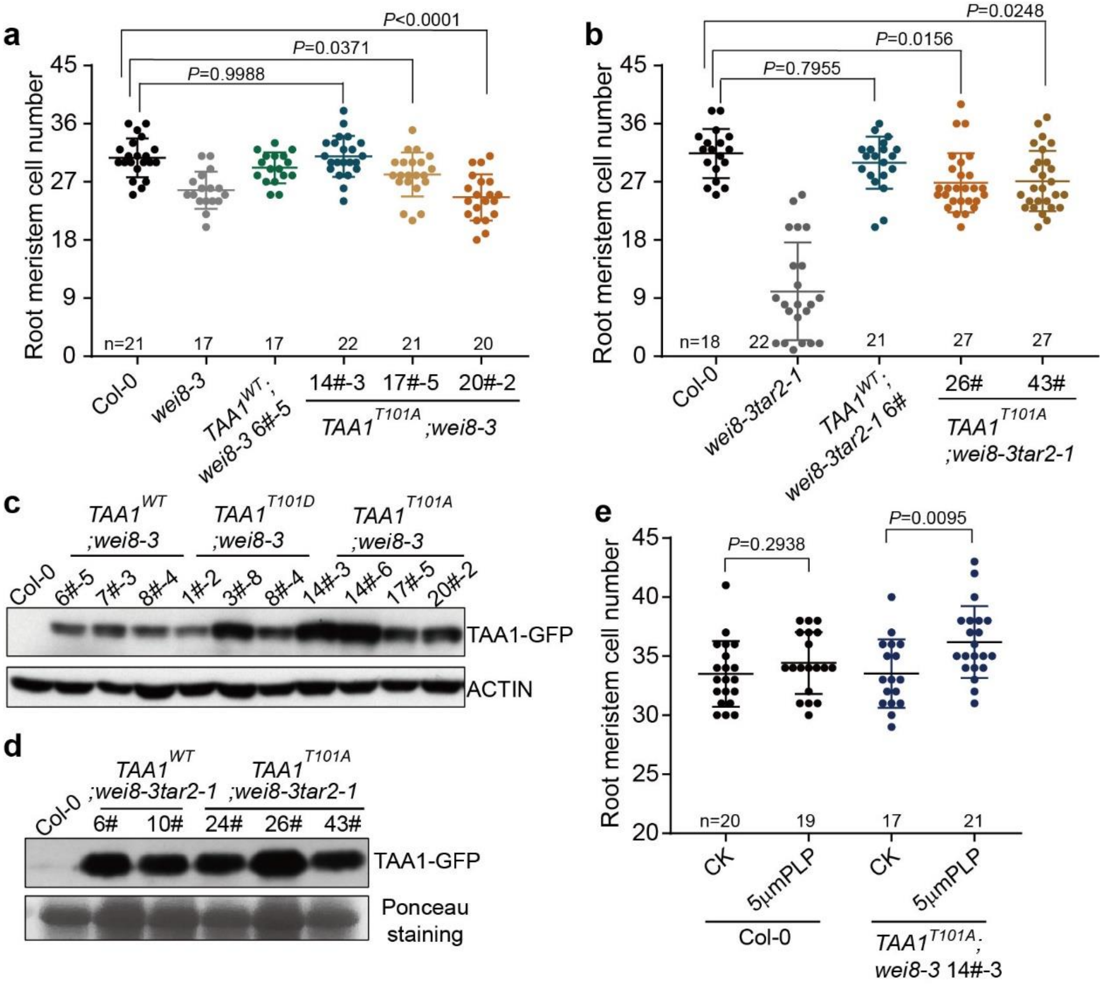

Supplementary Figure 5. T101A is not fully functional *in vivo*.

(**a**) Quantification of root meristem size of *pTAA1-TAA1*^{T101A}*;wei8-3* transgenic plants. n denotes number of independent seedlings. *P*-values were shown as indicated. One-way ANOVA with Dunnett multiple comparisons test was used comparing with Col-0. *P*<0.05 indicates a significant difference.

(**b**) Quantification of root meristem size of *pTAA1-TAA1*^{T101A}*;wei8-3;tar2-1* transgenic plants. n denotes number of independent seedlings. *P*-values were shown as indicated. One-way ANOVA with Dunnett multiple comparisons test was used comparing with Col-0. *P*<0.05 indicates a significant difference.

(**c, d**) Western blot showed TAA1-GFP protein levels of different transgenic plants used in (a) and (b). 5-day-old seedlings were used for phenotypic analyses and proteins extraction. TAA1-GFP proteins were detected by anti-GFP antibodies (HT801, TransGen Biotech).

(**e**) Quantification of root meristem size of *pTAA1-TAA1*^{T101A}*;wei8-3* treated with 5 μM PLP. 5-day-old seedlings grown on 1/2 MS plates with or without 5 μM PLP were used for phenotypic analysis. n denotes number of independent seedlings. *P*-values were shown as indicated. Two-sided *t*-test. Three independent repeats.

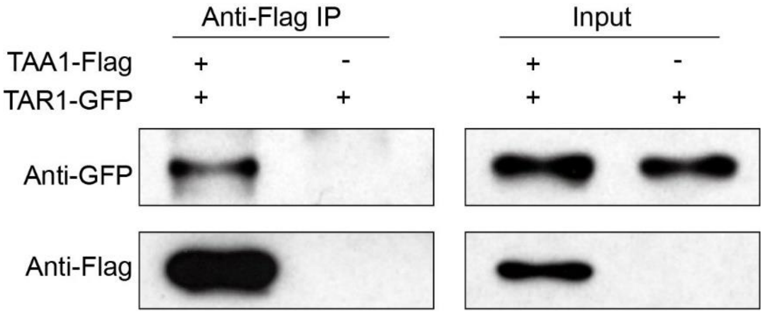

Supplementary Figure 6. TAA1 and TAR1 form heterodimers *in vivo*.

Co-IP assay showed interactions between TAA1 and TAR1 in Col-0 protoplast. 35S-TAA1-Flag and 35S-TAR1-GFP were transiently co-expressed in *Arabidopsis* protoplasts, single transformation of 35S-TAR1-GFP was used as a negative control. Three independent repeats with similar results.

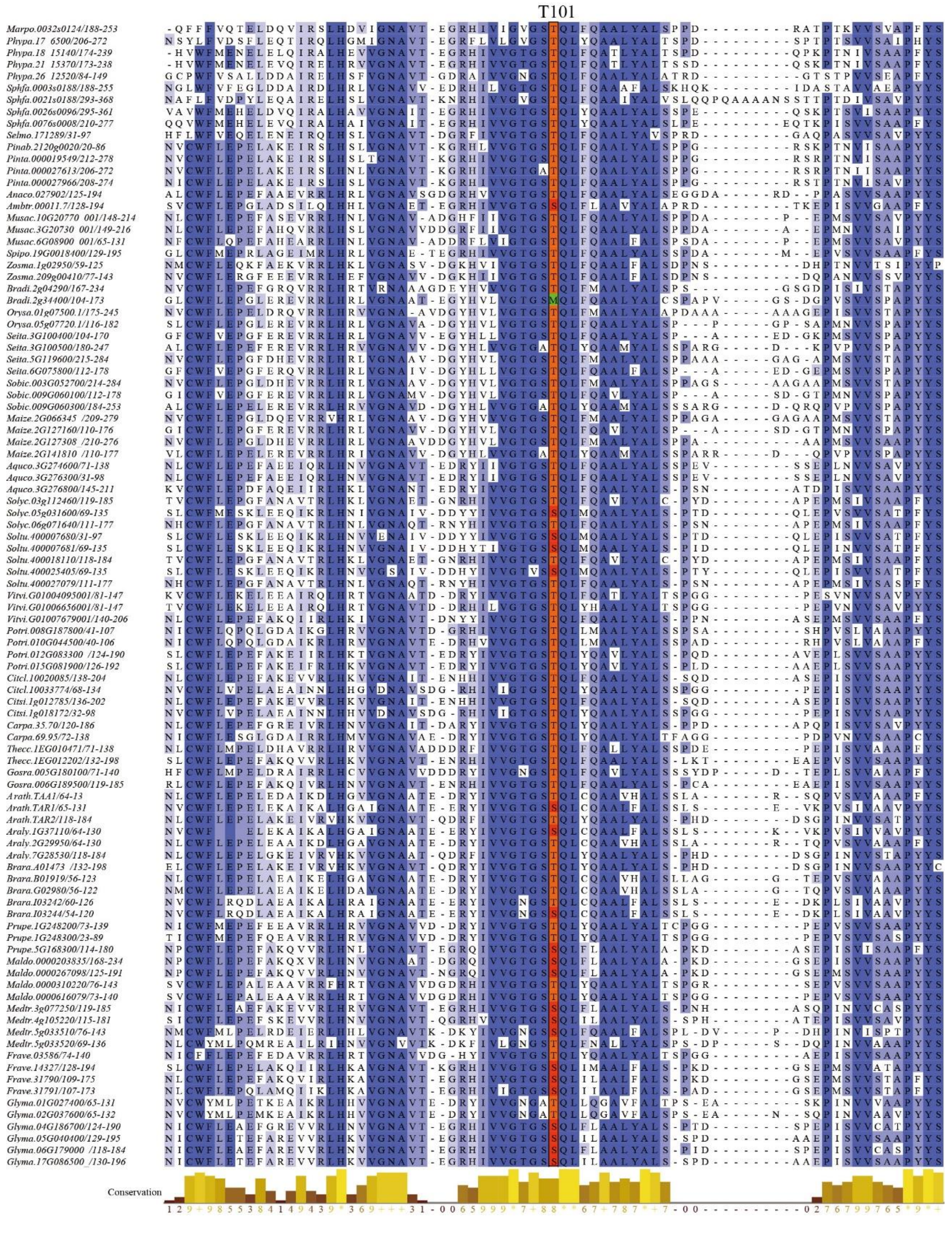

Supplementary Figure 7. Sequence alignment of TAA1 orthologs among 32 species. For detailed information about proteins and species, see Supplementary Table 1. T101 residue of AtTAA1 was highlighted in orange.

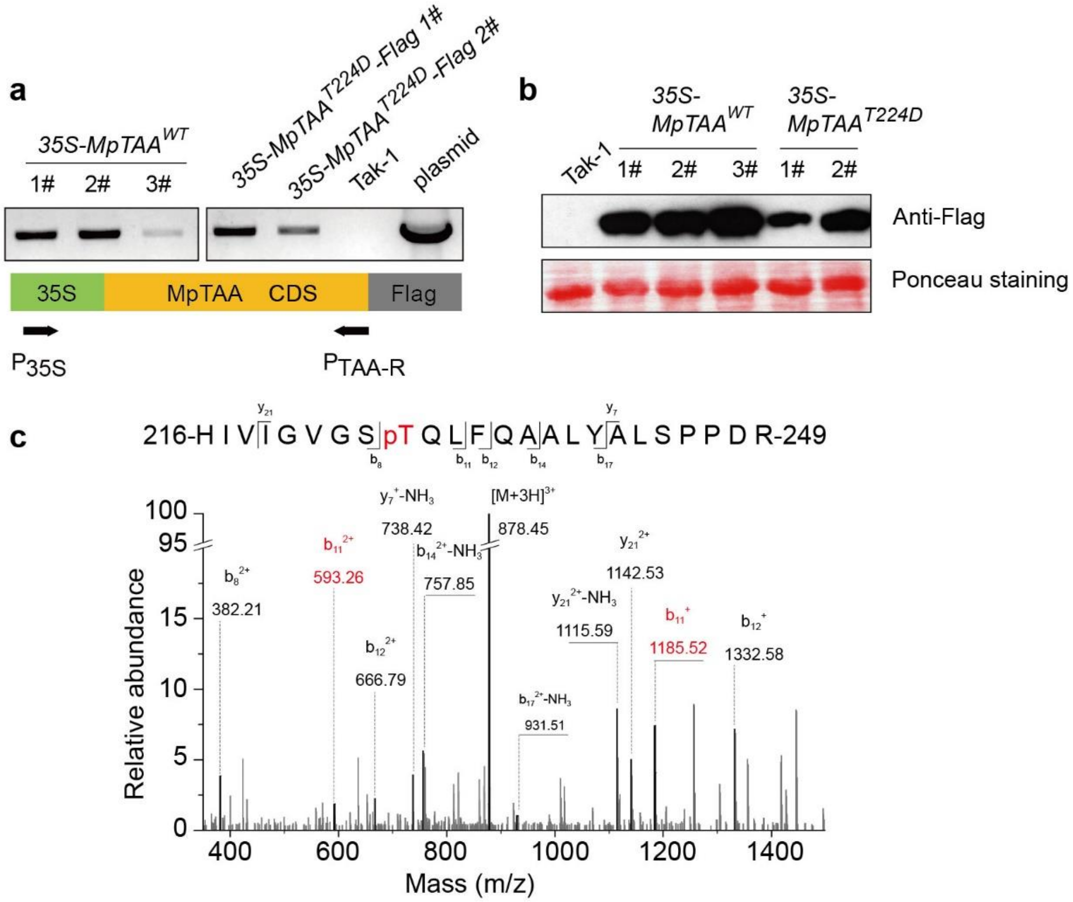

Supplementary Figure 8. Phosphorylation modification of Thr224 on MpTAA protein *in vivo*.

(**a**) PCR results showed successful transformation of constructs. 10-day-old thalli were used for genomic DNA extraction. Plasmid (35S-MpTAA-Flag) was used as a positive control. Arrows show the primers used for PCR. Primer sequences were list in Supplementary Table 2.

(**b**) Western blot results showed MpTAA-Flag protein levels of different transgenic plants. 20-day-old thalli were used for protein extraction. MpTAA-Flag proteins were detected by anti-Flag antibodies (M20008L, Abmart).

(**c**) Mass spectrometric analysis showed phosphorylation modification at Thr224 residue on MpTAA proteins. The mass increased 80 Da (weight of phosphate group) starting at b_{11} ion that defined the phosphorylation modification at Thr224. 3-week-old *35S-MpTAA-Flag* transgenic *Marchantia* was used to immuno-precipitate MpTAA proteins.

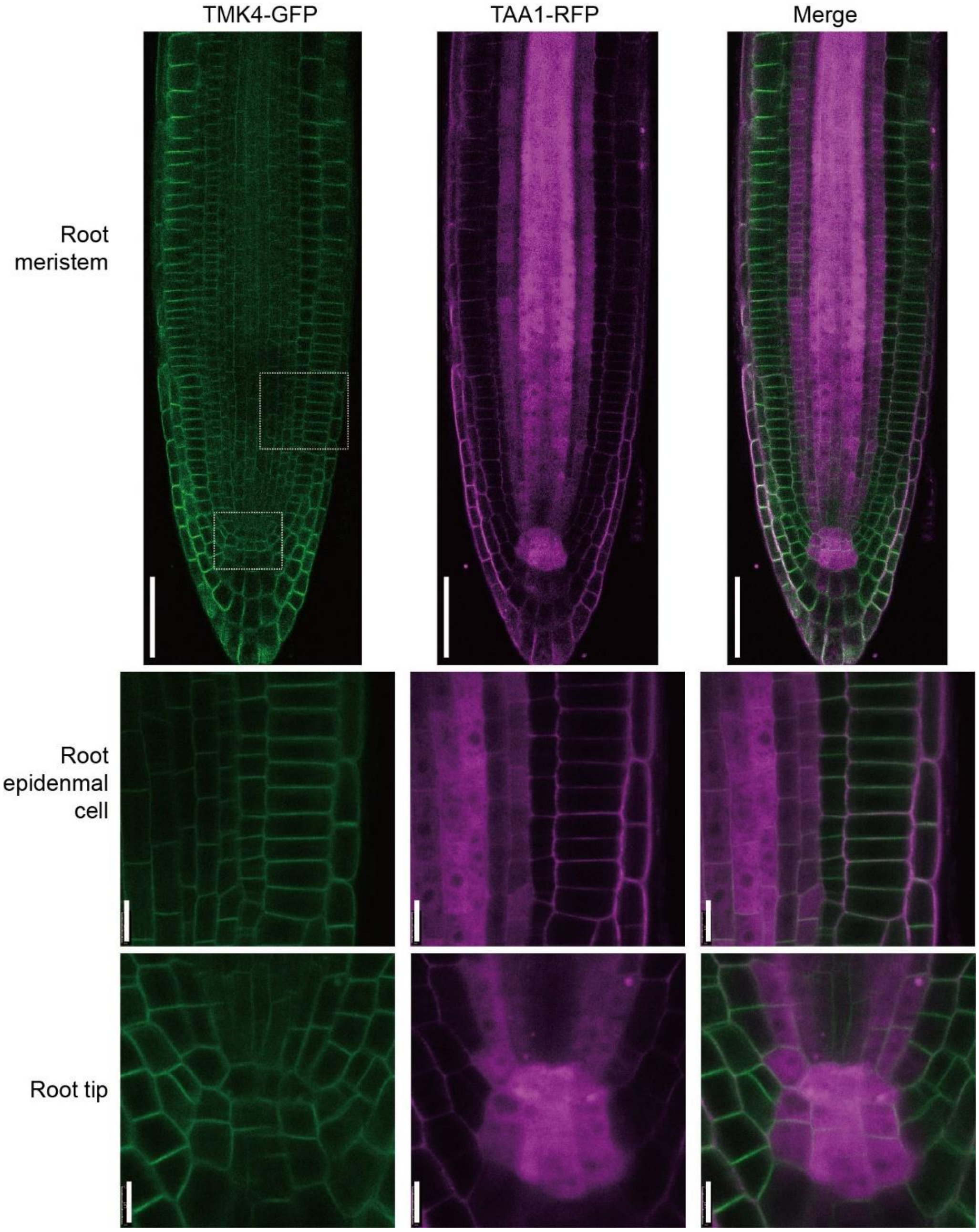

Supplementary Figure 9. Subcellular co-localization of TMK4 and TAA1 in roots.
The pictures were taken by TCS SP8 microscopy using 5-day-old transgenic plants (*pTMK4-TMK4-GFP* crossed with *pTAA1-TAA1-RFP*). About 7 individual seedlings were observed. The upper pictures showed whole root meristem zone, Scale bar, 50 μm. Boxes indicates two regions magnified to show root epidermal cells and root tip respectively. Scale bar, 8 μm.

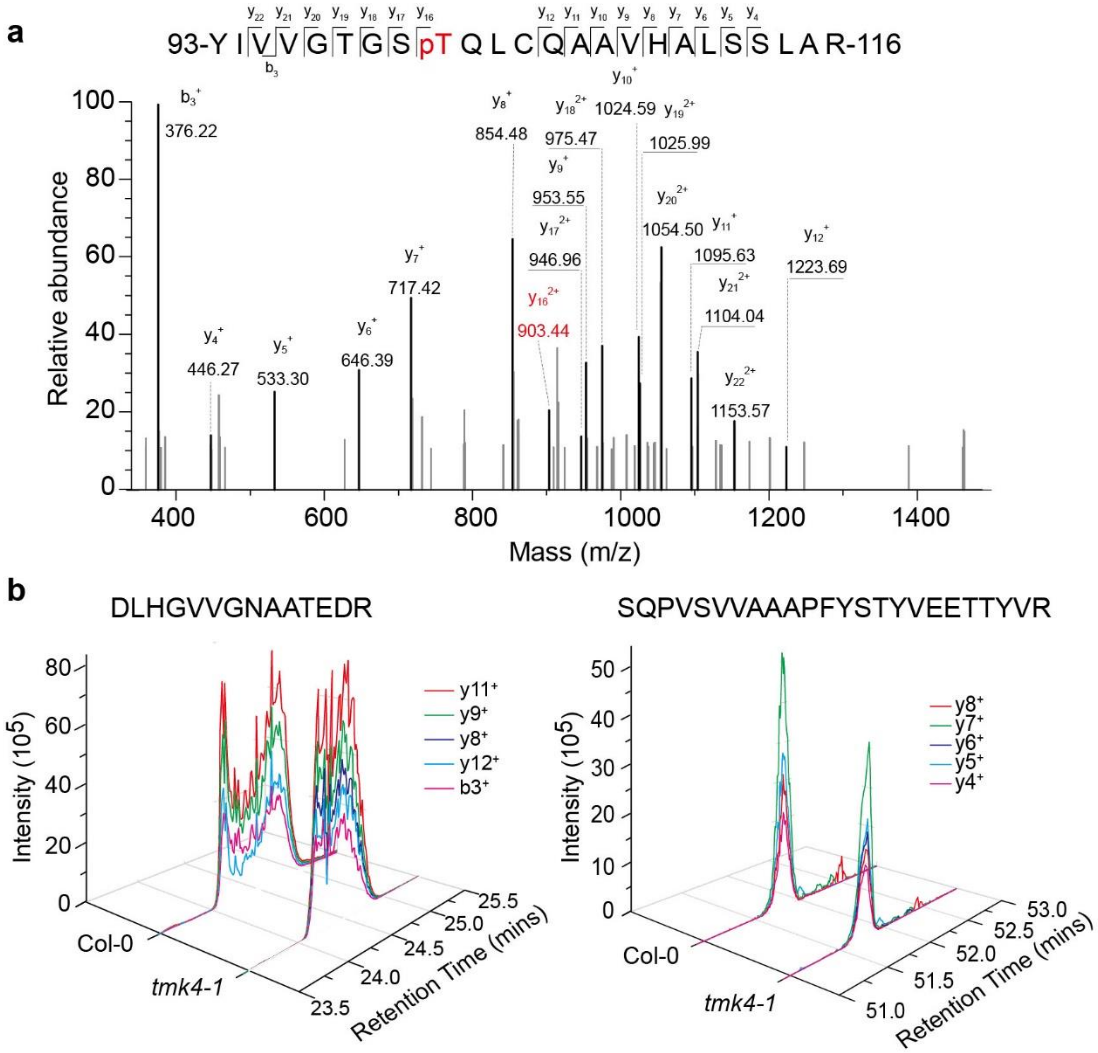

c

Replicates	Peptide	Area Ratio (*tmk4-1*/Col-0)
1	Non-phosphopeptides	0.63
	T101-phosphopeptides	0.28
2	Non-phosphopeptides	1.06
	T101-phosphopeptides	0.25
3	Non-phosphopeptides	0.56
	T101-phosphopeptides	0.35

Supplementary Figure 10. MS analyses showing TMK4 phosphorylates T101 site on TAA1 protein.

(**a**) Phosphor-peptide containing T101 site was detected by mass spectrometric analysis. *E.coli*-purified TAA1 proteins were used to perform the *in vitro* kinase reaction together

with TMK4 kinase domain then sent for MS analysis to identify phosphorylation sites. The mass increased 80 Da (weight of phosphate group) starting at y_{16} ion defined the phosphorylation modification at Thr101.

(**b**) Representative mass spectrometric analysis of two non-phosphorylation peptides indicated protein amount of TAA1-GFP in Col-0 and *tmk4-1*. This was shown as the internal reference of Fig.3e.

(**c**) Three independent biological repeats of mass spectrometric analysis showed T101 phosphorylation level of TAA1 decreased in *tmk4-1*.

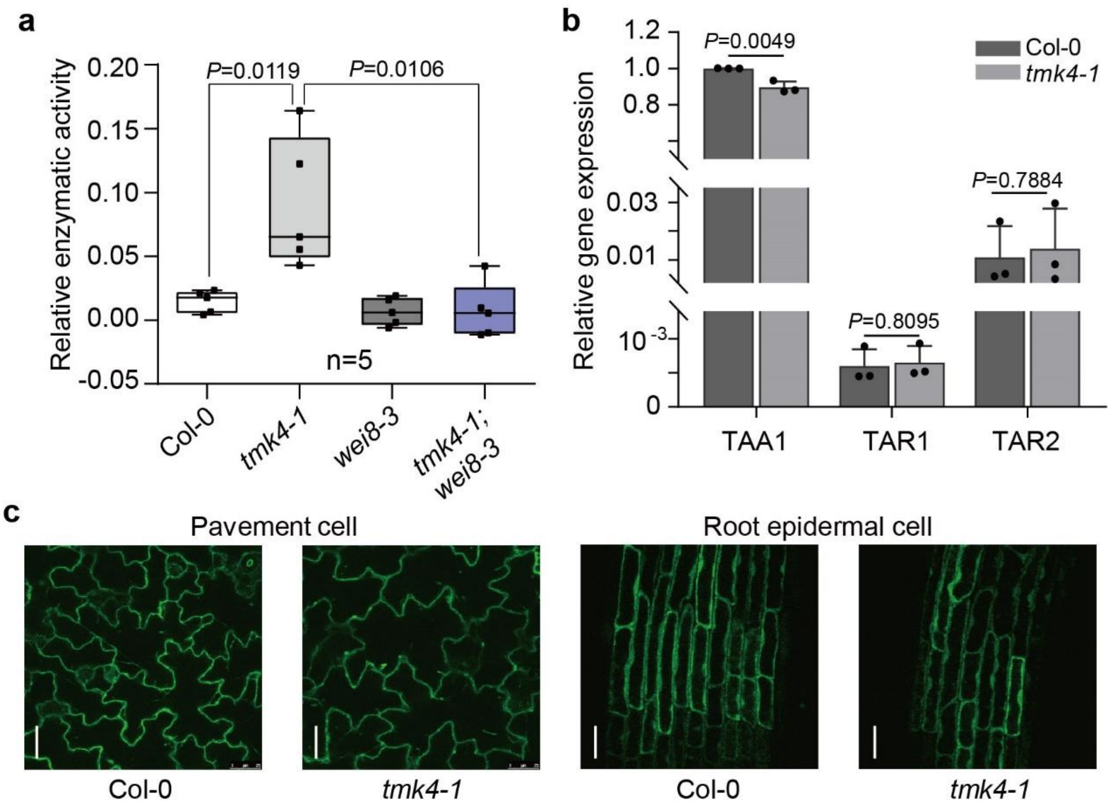

Supplementary Figure 11. TMK4 negatively regulates TAA1 enzymatic activity but not the transcription and subcellular localization of TAA1 *in vivo*.

(**a**) *In vivo* transaminase catalytic activity in Col-0, *tmk4-1*, *wei8-3* and *tmk4-1;wei8-3*. IPA concentration was determined with Salkowski reagent (see methods). Transaminase catalytic reactions were performed with reaction reagents containing protein extracts from Col-0, *tmk4-1*, *wei8-3* and *tmk4-1;wei8-3* roots (n=five biological repeats). *P*-values were shown as indicated, two-sided *t*-test. $P<0.05$ indicates a significant difference. Centre line in box represents mean and whiskers show minimum to maximum.

(**b**) qRT-PCR assay showed *TAA1* families gene expression level in Col-0 and *tmk4-1*. Roots from 5-day-old seedlings were used for RNA extraction. Data represented mean with s.d. from three biological repeats. *P*-values were shown as indicated, two-sided *t*-test.

(**c**) Subcellular localization of TAA1-GFP in Col-0 and *tmk4-1*. Images were taken from pavement cells and root epidermal cells. Scale bar, 25 μm.

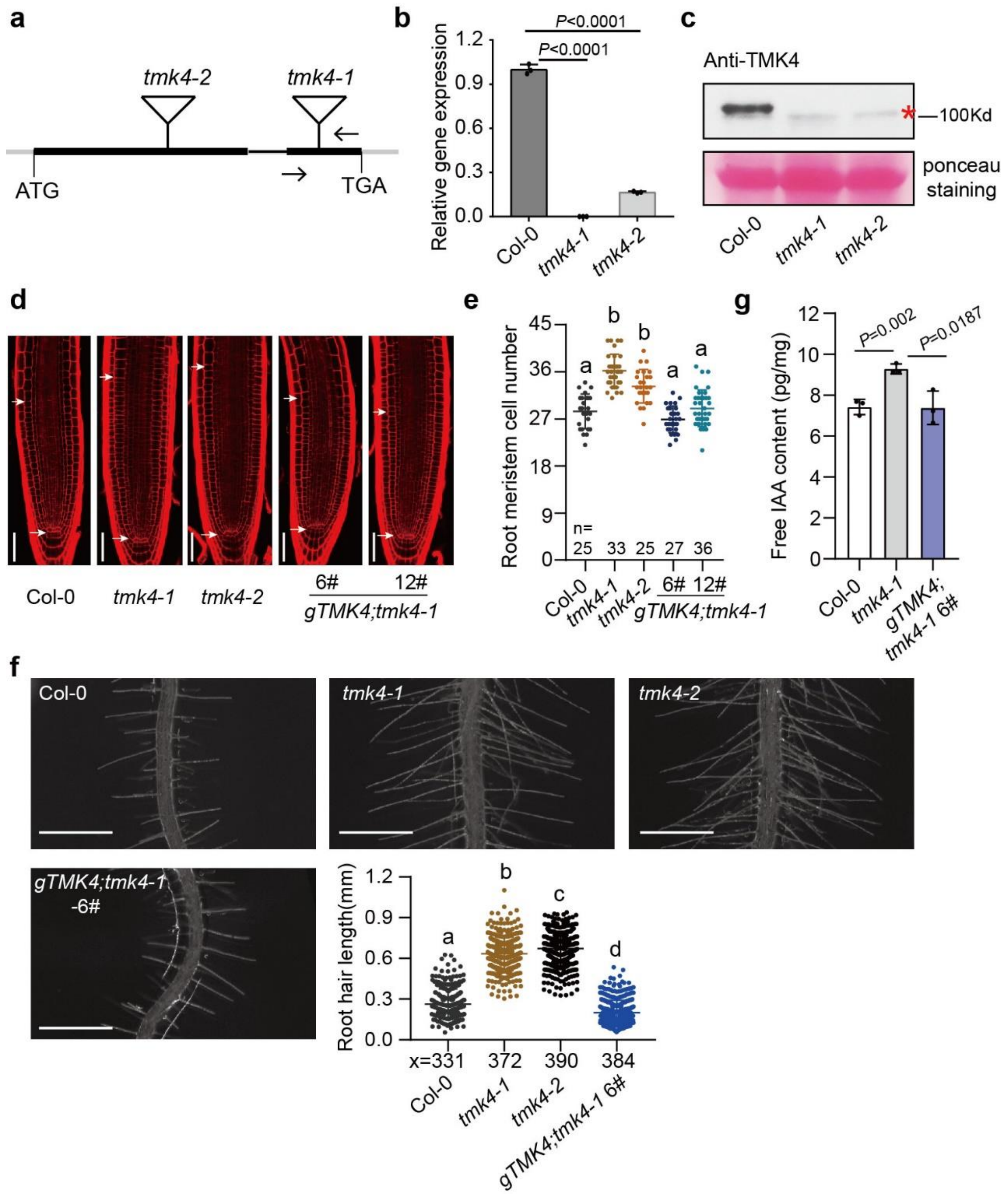

Supplementary Figure 12. TMK4 negatively regulates auxin content and root development-related processes.

(**a**) Description of T-DNA insertion mutants of *tmk4*. Lines represent introns; black and grey boxes represent exons and untranslated regions. Arrows show primers used for *TMK4* gene expression detection. Primer sequences were listed in Supplementary Table 2.

(**b**) qRT-PCR assay showed *TMK4* expression level in different *tmk4* mutants. Data represented mean with s.d., *P*-values were shown as indicated, two-sided *t*-test.
(**c**) Western blot results showed TMK4 protein level in *tmk4* mutants detected by TMK4 antibody. The red asterisk denotes a non-specific band.

(**d**) Representative images of root meristem from 5-day-old seedlings in *tmk4* mutants and *pTMK4-gTMK4-GFP;tmk4-1* complementation transgenic lines. White arrows show the meristem zone; scale bar, 50 μm.
(**e**) Quantification of root meristem size in (d**)**. n denotes number of independent seedlings. One-way ANOVA with Tukey multiple comparisons test was used. Different letters represent significant difference between each other, $P<0.0001$.
(f) Representative pictures of root hair from 5-day-old *tmk4* mutants and complementation transgenic lines (n=31) and quantification data. n denotes number of individual seedlings and x represents number of root hairs used for quantification. Scale bar, 0.5 mm. 3 independent complementation transgenic lines showed similar results. One-way ANOVA with Tukey multiple comparisons test was used. Different letters represent significant difference between each other, $P<0.0001$.
(**g**) Free IAA quantification of 8-day-old seedlings in *tmk4-1* and complementation transgenic line. Values indicated mean ± s.d. from three individual biological repeats. *P*-values were shown as indicated, two-sided *t*-test.

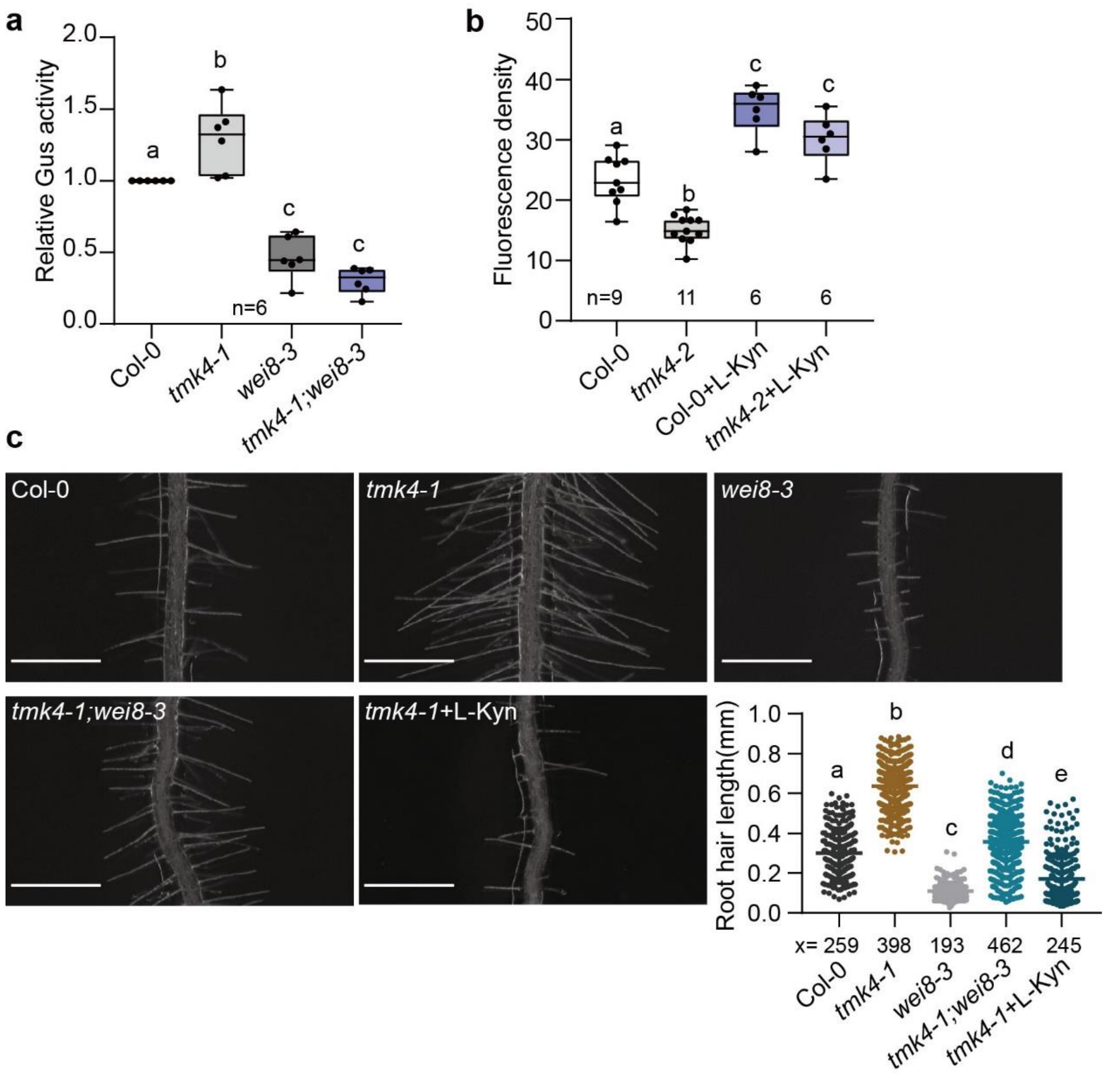

Supplementary Figure 13. TAA1 contributes to overproduced auxin in *tmk4-1*.

(**a**) Quantification data and statistical analysis of DR5-GUS activity in different mutants. Six independent biological repeats (n) were performed. Centre line in box represents mean and whiskers show minimum to maximum.

(**b**) Quantification of DII-Venus fluorescence signal in Fig. 4b. 1.5 μM L-Kyn was used for treatment. n denotes number of independent seedlings. Centre line in box represents mean and whiskers show minimum to maximum.

(**c**) Representative pictures of root hair from 5-day-old seedlings and quantification data. Col-0: n=20; *tmk4-1*: n=30; *wei8-3*: n=20; *tmk4-1;wei8-3*: n=34; *tmk4-1* +L-Kyn: n=19. n denotes number of independent seedlings and x represents number of root hairs used for quantification. Scale bar, 0.5 mm. One-way ANOVA with Tukey multiple

comparisons test was used in (a-c). Different letters represent significant difference between each other. $P<0.05$(a), $P<0.01$(b), $P<0.0001$(c).

a

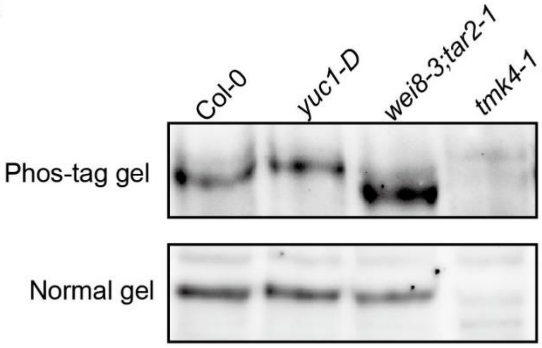

b

Replicates		Peptide	Area Ratio (Auxin / mock)	Phosphorylation up regulated ratio
1	Col-0	Non-phosphopeptides	0.902	**2.252**
		T101-phosphopeptides	2.031	
	tmk4-1	Non-phosphopeptides	1.110	**1.335**
		T101-phosphopeptides	1.482	
2	Col-0	Non-phosphopeptides	0.779	**2.239**
		T101-phosphopeptides	1.744	
	tmk4-1	Non-phosphopeptides	1.180	**1.009**
		T101-phosphopeptides	1.191	

Supplementary Figure 14. TMK4 is involved in self-regulation of auxin biosynthesis. (**a**) Gel shift assay showed TMK4 phosphorylation level increased in *yuc1-D* but decreased in *wei8-3;tar2-1*. 5-day-old seedlings were used for protein extraction. TMK4 proteins were detected by anti-TMK4 antibody. Two biological repeats with similar results.

(**b**) Auxin enhanced T101 phosphorylation of TAA1 partially through TMK4. TAA1-GFP proteins used for quantitative mass spectrometric analysis were immuno-precipitated from the protoplasts treated with EtOH (as mock) or 500 nM IAA (sigma I2886) for 10 mins. 35S-TAA1-GFP plasmids were transformed into Col-0 and *tmk4-1* protoplasts incubated for 8 hrs.

Supplementary Table 1: Information on TAA proteins of different species.

No.	Name of proteins	Gene ID in Phytozome database	Organism	protein size (aa)
1	Marpo.0032s0124	*Mapoly0032s0124*	*Marchantia polymorpha*	531
2	Phypa.17_6500	*Pp3c17_6500*	*Physcomitrella patens*	525
3	Phypa.18_15140	*Pp3c18_15140*	*Physcomitrella patens*	519
4	Phypa.21_15370	*Pp3c21_15370*	*Physcomitrella patens*	511
5	Phypa.26_12520	*Pp3c26_12520*	*Physcomitrella patens*	442
6	Sphfa.0003s0188	*Sphfalx0003s0188*	*Sphagnum fallax*	551
7	Sphfa.0021s0188	*Sphfalx0021s0188*	*Sphagnum fallax*	659
8	Sphfa.0026s0096	*Sphfalx0026s0096*	*Sphagnum fallax*	669
9	Sphfa.0076s0008	*Sphfalx0076s0008*	*Sphagnum fallax*	559
10	Selmo.171289	*171289*	*Selaginella moellendorffii*	359
11	Pinab.2120g0020	*MA_2120g0020*	*Pinus abies*	352
12	Pinta.000019549	*PITA_000019549-RA*	*Pinus taeda*	565
13	Pinta.000027613	*PITA_000027613-RA*	*Pinus taeda*	538
14	Pinta.000027966	*PITA_000027966-RA*	*Pinus taeda*	541
15	Anaco.027902	*Aco027902*	*Ananas comosus*	433
16	Ambtr.00011.7	*evm_27.TU.AmTr_v1.0_ scaffold00011.7*	*Amborella trichopoda*	460
17	Musac.3G20730_001	*GSMUA_Achr3G20730_ 001*	*Musa acuminata*	468
18	Musac.6G08900_001	*GSMUA_Achr6G08900_ 001*	*Musa acuminata*	387
19	Musac.10G20770_00 1	*GSMUA_Achr10G20770 _001*	*Musa acuminata*	448
20	Spipo.19G0018400	*Spipo19G0018400*	*Spirodela polyrhiza*	450
21	Zosma.1g02950	*Zosma1g02950*	*Zostera marina*	383
22	Zosma.209g00410	*Zosma209g00410*	*Zostera marina*	403
23	Bradi.2g04290	*Bradi2g04290*	*Arabidopsis lyrata*	498
24	Bradi.2g34400	*Bradi2g34400*	*Brachypodium distachyon*	431

25	Orysa.01g07500.1	*LOC_Os01g07500.1*	*Oryza sativa*	507
26	Orysa.05g07720.1	*LOC_Os05g07720.1*	*Oryza sativa*	441
27	Seita.3G100400	*Seita.3G100400*	*Setaria italica*	424
28	Seita.3G100500	*Seita.3G100500*	*Setaria italica*	506
29	Seita.5G119600	*Seita.5G119600*	*Setaria italica*	540
30	Seita.6G075800	*Seita.6G075800*	*Setaria italica*	433
31	Sobic.003G052700	*Sobic.003G052700*	*Sorghum bicolor*	538
32	Sobic.009G060100	*Sobic.009G060100*	*Sorghum bicolor*	432
33	Sobic.009G060300	*Sobic.009G060300*	*Sorghum bicolor*	515
34	Maize.2G066345	*GRMZM2G066345*	*Zea mays*	536
35	Maize.2G127160	*GRMZM2G127160*	*Zea mays*	431
36	Maize.2G127308	*GRMZM2G127308*	*Zea mays*	530
37	Maize.2G141810	*GRMZM2G141810*	*Zea mays*	435
38	Aquco.3G274600	*Aqcoe3G274600*	*Aquilegia coerulea*	391
39	Aquco.3G276300	*Aqcoe3G276300*	*Aquilegia coerulea*	351
40	Aquco.3G276800	*Aqcoe3G276800*	*Aquilegia coerulea*	467
41	Solyc.03g112460	*Solyc03g112460.2*	*Solanum lycopersicum*	444
42	Solyc.05g031600	*Solyc05g031600.1*	*Solanum lycopersicum*	391
43	Solyc.06g071640	*Solyc06g071640.2*	*Solanum lycopersicum*	437
44	Soltu.400007680	*PGSC0003DMG400007680*	*Solanum tuberosum*	355
45	Soltu.400007681	*PGSC0003DMG400007681*	*Solanum tuberosum*	393
46	Soltu.400018110	*PGSC0003DMG400018110*	*Solanum tuberosum*	443
47	Soltu.400025405	*PGSC0003DMG400025405*	*Solanum tuberosum*	385
48	Soltu.400027079	*PGSC0003DMG400027079*	*Solanum tuberosum*	439
49	Vitvi.G01004095001	*GSVIVG01004095001*	*Vitis vinifera*	410
50	Vitvi.G01006656001	*GSVIVG01006656001*	*Vitis vinifera*	419
51	Vitvi.G01007679001	*GSVIVG01007679001*	*Vitis vinifera*	473

52	Potri.008G187800	*Potri.008G187800*	*Populus trichocarpa*	377
53	Potri.010G044500	*Potri.010G044500*	*Populus trichocarpa*	365
54	Potri.012G083300	*Potri.012G083300*	*Populus trichocarpa*	457
55	Potri.015G081900	*Potri.015G081900*	*Populus trichocarpa*	457
56	Citsi.1g012785	*orange1.1g012785m.g*	*Citrus sinensis*	456
57	Citsi.1g018172	*orange1.1g018172m.g*	*Citrus sinensis*	360
58	Citcl.10020085	*Ciclev10020085m.g*	*Citrus clementina*	458
59	Citcl.10033774	*Ciclev10033774m.g*	*Citrus clementina*	434
60	Carpa.35.70	*evm.TU.supercontig_35.70*	*Carica papaya*	448
61	Carpa.69.95	*evm.TU.supercontig_69.95*	*Carica papaya*	364
62	Thecc.1EG010471	*Thecc1EG010471*	*Theobroma cacao*	399
63	Thecc.1EG012202	*Thecc1EG012202*	*Theobroma cacao*	452
64	Gosra.005G180100	*Gorai.005G180100*	*Gossypium raimondii*	401
65	Gosra.006G189500	*Gorai.006G189500*	*Gossypium raimondii*	438
66	Arath.TAA1	*Arabidopsis TAA1*	*Arabidopsis thaliana*	391
67	Arath.TAR1	*Arabidopsis TAR1*	*Arabidopsis thaliana*	388
68	Arath.TAR2	*Arabidopsis TAR2*	*Arabidopsis thaliana*	440
69	Araly.1G37110	*AL1G37110*	*Arabidopsis lyrata*	387
70	Araly.2G29950	*AL2G29950*	*Arabidopsis lyrata*	392
71	Araly.7G28530	*AL7G28530*	*Arabidopsis lyrata*	440
72	Brara.A01473	*Brara.A01473*	*Brassica rapa*	454
73	Brara.B01919	*Brara.B01919*	*Brassica rapa*	381
74	Brara.G02980	*Brara.G02980*	*Brassica rapa*	381
75	Brara.I03242	*Brara.I03242*	*Brassica rapa*	381
76	Brara.I03244	*Brara.I03244*	*Brassica rapa*	382
77	Prupe.1G248200	*Prupe.1G248200*	*Prunus persica*	408
78	Prupe.1G248300	*Prupe.1G248300*	*Prunus persica*	354
79	Prupe.5G168300	*Prupe.5G168300*	*Prunus persica*	437
80	Maldo.0000203835	*MDP0000203835*	*Malus domestica*	431
81	Maldo.0000267098	*MDP0000267098*	*Malus domestica*	448

82	Maldo.0000310220	*MDP0000310220*	*Malus domestica*	404
83	Maldo.0000616079	*MDP0000616079*	*Malus domestica*	407
84	Medtr.3g077250	*Medtr3g077250*	*Medicago truncatula*	441
85	Medtr.4g105220	*Medtr4g105220*	*Medicago truncatula*	437
86	Medtr.5g033510	*Medtr5g033510*	*Medicago truncatula*	401
87	Medtr.5g033520	*Medtr5g033520*	*Medicago truncatula*	394
88	Frave.03586	*gene03586.1-v1.0-hybrid*	*Fragaria vesca*	434
89	Frave.31790	*gene31790.1-v1.0-hybrid*	*Fragaria vesca*	432
90	Frave.31791	*gene31791.1-v1.0-hybrid*	*Fragaria vesca*	530
91	Glyma.01G027400	*Glyma.01G027400*	*Glycine max*	391
92	Glyma.02G037600	*Glyma.02G037600*	*Glycine max*	392
93	Glyma.04G186700	*Glyma.04G186700*	*Glycine max*	445
94	Glyma.05G040400	*Glyma.05G040400*	*Glycine max*	452
95	Glyma.06G179000	*Glyma.06G179000*	*Glycine max*	439
96	Glyma.17G086500	*Glyma.17G086500*	*Glycine max*	453

Supplementary Table 2: Primers list.

Primers name	Primers sequence (5`-3`)	Notes
gTMK4 GW-F	GGGGACAAGTTTGTACAAAAAAGCAGGCTTATGTGGTTTTAGTCATTTATT	PDONR-zeo construction
gTMK4 GW-R	GGGGACCACTTTGTACAAGAAAGCTGGGTAACCATCAGCTGAATCGAAAGTA	
gTAA1 GW-F	GGGGACAAGTTTGTACAAAAAAGCAGGCTTACTCCATATCGAGGGTATGAA	
gTAA1 GW-R	GGGGACCACTTTGTACAAGAAAGCTGGGTAAAGGTCAATGCTTTTAA	
TAA1 GW-F	GGGGACAAGTTTGTACAAAAAAGCAGGCTTAATGGTGAAACTGGAGAACTC	
TAA1 GW-R	GGGGACCACTTTGTACAAGAAAGCTGGGTAAAGGTCAATGCTTTTAA	
MpTAA GW-F	GGGGACAAGTTTGTACAAAAAAGCAGGCTTAATGTCGTCTCGAATGGACTCA	
MpTAA GW-R	GGGGACCACTTTGTACAAGAAAGCTGGGTACGACATGTCGACTGCAGACTG	
tmk4-1 LP	TGCGATTGCTCAAAGAGGTCAGA	Genotyping primers
tmk4-1 RP	GGCTGCATTGGTTGCACTGGAT	
tmk4-2 LP	AAATGTCTATGCCTGACC	
tmk4-2 RP	TCCAAGAGCGAGAATCTC	
wei8-3 LP	CATGATGTCATGGTCTTGACG	
wei8-3 RP	TTTACTCCGTAAGTCCCCACC	
tar2-1 LP	CACAATCTCTTTGGCAAGCTC	
tar2-1 RP	TAGTGGAATTTTTGATTGCTTG	
tar1-1 LP	GATTCGTTATGACATTTGGCG	
tar1-1 RP	CGATTGAAGATACGATGCCTC	
P35S	GACGCACAATCCCACTATCC	
PTAA-R	CTACGACATGTCGACTGC	
TMK4 qRT-F	AGGCACTCGACCAAACCTTA	qRT-PCR primers
TMK4 qRT-R	ACGACGGTTTCCACTTCTCT	
TAA1 qRT-F	GATGAAGAATCGGTGGGAGA	
TAA1 qRT-R	TGACTCGGACATGCTTCTTG	
TAR1 qRT-F	TCCATTGGTGTGTCGAAGGA	
TAR1 qRT-R	AAACGCAGGAGAAGTGGAGA	
TAR2 qRT-F	GCTCTTCACTGCTTCAAAGAGCAC	
TAR2 qRT-R	TCTGTCTTTCACCAAAGCCCATCC	
UBQ10 qRT-F	GATCTTTGCCGGAAAACA	
UBQ10 qRT-R	CGAAGATGAGACGCTGCT	
PINOID NdeI F	GGCATTCCATATGATGTTACGAGAATCAGAC	pBKT7-vector construction
PINOID EcoRI R	CCGGAATTCAAAGTAATCGAACGCCGCTGG	
D6PK NdeI F	GGCATTCCATATGATGATGGCTTCAAAAACTCC	
D6PK PstI R	CCGCTGCAGGGAAGAAATCAAACTCAAGA	
FLS2 C NdeI F	GGCATTCCATATGAATTCATCAGAGTCCTCATT	

FLS2 C BamHI R	AGGTCGACGGATCCCAACTTCTCGATCCTCGTTACG	
BAK1 C NdeI F	GGCATTCCATATGTTCTTTGATGTACCAGCTGA	
BAK1 C BamHI R	CGCGGATCCTTATCTTGGACCCGAGGGGT	
BIN2 NdeI F	GGCATTCCATATGATGGCTGATGATAAGGAGAT	
BIN2 EcoRI R	CCGGAATTCAGTTCCAGATTGATTCAAGAA	
BRI1 C NdeI F	GGCATTCCATATGTTCCATAATGATAGTCTGAT	
BRI1 C EcoRI R	CCGGAATTCTCATAATTTTCCTTCAGGAA	
TAA1 XmaI R	TCGACCCGGGCTAAAGGTCAATGCTTTT	pGEX4T-2 vector construction
TAA1 BamHI F	CGCGGATCCATGGTGAAACTGGAGAACTC	
TAA1 StuI R	AAAAGGCCTAAGGTCAATGCTTTTAATGA	HBT vector construction
TMK4 KD EcoRI F	ATCCGAATTCTTCAGTGAGGATAACATA	His-sumo vector/ PGEX4T-2 vector construction
TMK4 KD NotI R	ATTATGCGGCCGCTCACCGACCATCAGCTG	
TMK4 KD BmaHI	CCGCGTGGATCCGGGGGTTTCGGTGTCGTG	PET14 vector and HBT vector construction
TMK4 BclI F	GCGCCTGATCAATGGAGGCTCCTACGCCTC	
TMK4 StuI R	AAAAGGCCTCCGACCATCAGCTGAATCGA	Site-directed mutagenesis
TAA1 T101A F	AGTGGTTGGGACCGGTTCGGCGCAGCTTTGTCAAGC	
TAA1 T101A R	GCTTGACAAAGCTGCGCCGAACCGGTCCCAACCACT	
TAA1 T101D F	GGTTGGGACCGGTTCGGACCAGCTTTGTCAAGCCG	
TAA1 T101D R	CGGCTTGACAAAGCTGGTCCGAACCGGTCCCAACC	
MpTAA T224D F	TCCGACCAACTCTTCCAGGCTGCATTG	
MpTAA T224D R	CAATGCAGCCTGGAAGAGTTGGTCGGA	
