# Supplementary material for: A phosphorylation-based switch controls TAA1-mediated auxin biosynthesis in plants
Source: Nat Commun. 2020 Feb 3;11:679. doi: 10.1038/s41467-020-14395-w (PMC6997161; doi:10.1038/s41467-020-14395-w)
Supplement: Supplementary file 3 — Reporting Summary [file 41467_2020_14395_MOESM3_ESM.pdf]

natureresearch

Corresponding author(s): Tongda Xu

Reporting Summary

Nature Research wishes to improve the reproducibility of the work that we publish. This form provides structure for consistency and transparency in reporting. For further information on Nature Research policies, see Authors & Referees and the Editorial Policy Checklist.

Statistical parameters

When statistical analyses are reported, confirm that the following items are present in the relevant location (e.g. figure legend, table legend, main text, or Methods section).

n/a	Confirmed	
☐	☒	The exact sample size (*n*) for each experimental group/condition, given as a discrete number and unit of measurement
☐	☒	An indication of whether measurements were taken from distinct samples or whether the same sample was measured repeatedly
☐	☒	The statistical test(s) used AND whether they are one- or two-sided<br>*Only common tests should be described solely by name; describe more complex techniques in the Methods section.*
☒	☐	A description of all covariates tested
☐	☒	A description of any assumptions or corrections, such as tests of normality and adjustment for multiple comparisons
☐	☒	A full description of the statistics including central tendency (e.g. means) or other basic estimates (e.g. regression coefficient) AND variation (e.g. standard deviation) or associated estimates of uncertainty (e.g. confidence intervals)
☐	☒	For null hypothesis testing, the test statistic (e.g. *F*, *t*, *r*) with confidence intervals, effect sizes, degrees of freedom and *P* value noted<br>*Give P values as exact values whenever suitable.*
☒	☐	For Bayesian analysis, information on the choice of priors and Markov chain Monte Carlo settings
☒	☐	For hierarchical and complex designs, identification of the appropriate level for tests and full reporting of outcomes
☒	☐	Estimates of effect sizes (e.g. Cohen's *d*, Pearson's *r*), indicating how they were calculated
☐	☒	Clearly defined error bars<br>*State explicitly what error bars represent (e.g. SD, SE, CI)*

Our web collection on statistics for biologists may be useful.

Software and code

Policy information about availability of computer code

Data collection	Proteomic data was collected by Orbitrap Fusion (Thermo Fisher Scientific, Watham, MA), by software Thermo Xcalibur 3.0.63.
Data analysis	Proteomic analysis: Mascot Daemon 2.5.0.100(64-bit) (Matrix Science, London, UK);Skyline (version529 4.1.0.18163)<br>Alignments: Geneious® 10.2.3<br>Statistical analysis: GraphPad Prism 7<br>Image analysis: ImageJ 1.8.0 and Quantity One v462

For manuscripts utilizing custom algorithms or software that are central to the research but not yet described in published literature, software must be made available to editors/reviewers upon request. We strongly encourage code deposition in a community repository (e.g. GitHub). See the Nature Research guidelines for submitting code & software for further information.

Data

Policy information about availability of data

All manuscripts must include a data availability statement. This statement should provide the following information, where applicable:

- Accession codes, unique identifiers, or web links for publicly available datasets
- A list of figures that have associated raw data
- A description of any restrictions on data availability

The data supporting the findings in this study are available and described within the paper and its supplemental information. Source Data (gels and graphs) for Figs. 1- 4 and supplementary Figs. 2-6, 8, 10-14 are provided as a separate Source Data file.

Field-specific reporting

Please select the best fit for your research. If you are not sure, read the appropriate sections before making your selection.

[x] Life sciences [ ] Behavioural & social sciences

For a reference copy of the document with all sections, see nature.com/authors/policies/ReportingSummary-flat.pdf

Life sciences

Study design

All studies must disclose on these points even when the disclosure is negative.

Sample size	Sample size was determined by previous publications. These were clearly indicated in each figure legends or methods.
Data exclusions	No data was excluded.
Replication	All attempts of replication by different people within the group were successful.
Randomization	The samples were picked to do biochemistry assay is randomized; seedlings with similar growth condition were used for root phenotypic analyses.
Blinding	The key phenotype analysis was confirmed by double blind. We did not do blinding assay to biochemistry data because the loading of gel need a known sample order.

Materials & experimental systems

Policy information about availability of materials

n/a	Involved in the study
[ ]	[x] Unique materials
[ ]	[x] Antibodies
[x]	[ ] Eukaryotic cell lines
[x]	[ ] Research animals
[x]	[ ] Human research participants

Unique materials

Obtaining unique materials	All unique materials used are readily available from the authors, and can be requested from corresponding author by tdxu@sibs.ac.cn or txu002@ucr.edu

Antibodies

Antibodies used	Commercial antibodies used:<br>α-GFP (HT801, TransGen Biotech, 1:2000 dilution), α-HA-HRP (HA-7, #H6533, Sigma, 1:2000 dilution), α-Flag (# M20008L , Abmart, 1:2000 dilution), α-Actin (# M20009L , Abmart, 1:2000 dilution), α-GST(#M20007, Abmart, 1:2000 dilution). Expect α-HA-HRP, other primary antibodies were detected using Goat Anti-Mouse IgG (H+L)-HRP Conjugate (#1721011, Bio-Rad, 1:5000 dilution).<br>α-TMK4 was produced by ABclonal Biotechnology and used 1:2000 dilution for Western blot. α-TMK4 primary antibodies were detected using Goat Anti-Rabbit IgG (H + L)-HRP Conjugate.(#1706515, Bio-Rad, 1:2000 dilution)
Validation	Validation statements of commercial primary antibodies are available from manufacturers: α-GFP, Transgen(http://www.transgen.com.cn/data/upload/pdf/HT801_2019-11-05.pdf), α-HA-HRP(https://www.sigmaaldrich.com/content/dam/sigma-aldrich/docs/Sigma/Datasheet/2/h6533dat.pdf), α-Flag(www.ab-mart.com.cn/upload/20170614093631xz.pdf), α-Actin (www.ab-mart.com.cn/upload/20170614135558xz.pdf), α-GST(http://www.ab-mart.com.cn/upload/20180315105109xz.pdf).

α-TMK4 was validated by western blot in the tmk4 mutant (Supplementary Fig. 12c)

Method-specific reporting

n/a	Involved in the study
☒	☐ ChIP-seq
☒	☐ Flow cytometry
☒	☐ Magnetic resonance imaging
